# Experimental and simulation study of American saffron seed oil blended with diesel

**DOI:** 10.1016/j.heliyon.2024.e34959

**Published:** 2024-07-23

**Authors:** Valiveti Sivaramakrishna, Shaik Hussain, Chintalapudi Ravi Kiran, Jayashri N. Nair, Irfan Anjum Badruddin, Abdul Saddique Shaik, Sarfaraz Kamangar, Muhammad Mahmood Ali, Muhammad Nasir Bashir

**Affiliations:** a Department of Mechanical Engineering, VNR Vignana Jyothi Institute of Engineering and Technology, Hyderabad, Telangana, 500090, India; b Malla Reddy Engineering College(Autonomous), Hyderabad, Telangana, India, 500100; c Department of Mechanical Engineering, MLR Institute Of Technology, Dundigal, Hyderabad, Telangana, 500043, India; d Mechanical Engineering Department, College of Engineering, King Khalid University, Abha, 61421, Saudi Arabia; e Department of Mechatronic Engineering, Atlantic Technological University Sligo, Ash Lane, F91 YW50 Sligo, Ireland; f Department of Mechanical Engineering, Yonsei University, Seoul, 120-749, Republic of Korea

**Keywords:** American saffron oil methyl ester, DIESEL-RK, Performance, Emission, Diesel engine

## Abstract

In a variety of industries, including transportation, agriculture, and manufacturing, diesel engines are often employed. Due of rising prices and environmental concerns, researchers examined whether biodiesels might replace diesel. The current study looks into American Saffron Oil's feasibility as a feedstock for biodiesel production. The transesterification technique is used to extract American saffron oil methyl este(ASOME), which is then examined for its physical and chemical properties in accordance with ASTM standards. Diesel fuel and American Saffron Oil methyl ester are mixed on a volume basis to create a variety of fuel blends, including B20, B40, and B60. The test results showed that the 20 % ASOME mix had better performance and reduced emissions. Also, utilizing DIESEL-RK simulation software, diesel engine tests are conducted for the B20, B40, and B60 under identical operating circumstances. Moreover, diesel engine testing for the B20, B40, and B60 are carried out using DIESEL-RK simulation software under comparable operating circumstances. Results of simulation software tests show improved engine performance and reduced pollutants. When experimental data is compared to DIESEL-RK modeling software, it is found that brake thermal efficiency increased by 5.7 % and emissions of hydrocarbon and carbon monoxide decreased by 2.5 % and 14.3 %, respectively.

## Graphical abstract

**Figure:**
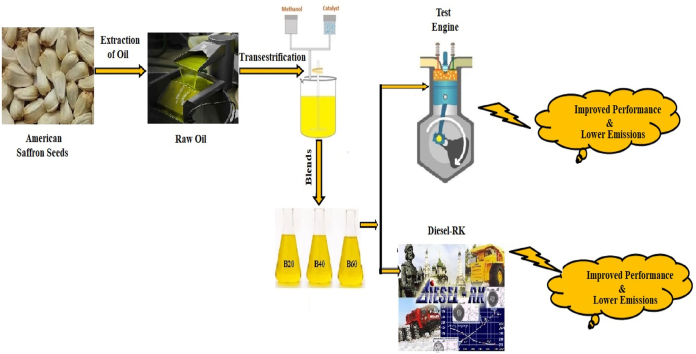

## 1 Introduction

The transportation sector contributes significantly to air pollution, and its expansion is having increasing negative environmental impacts. 13 of the 20 cities on the WHO's list of the world's 20 most polluted cities in 2014 were in India. In India's major cities, air pollution levels are rising quickly. Moreover, increased efforts to protect the environment have increased research into green fuels such biofuels [Kolakoti] [1]. The ratio of the various lipids and compounds determines the properties that the biofuels contain. Hence, the characteristics of the available seeds are determined before they are transesterified into biodiesel.

When non-edible oil is used as a fuel instead of diesel, the creation of biodiesel is more favorable. The usage of edible oil will affect the food industry and raise the price of food and fuel. Diesel-RK simulation software is used to examine the tests done using polanga oil and the outcomes. B10 improved the thermal efficiency of the brakes. Sulfur dioxide (SO2) and nitrogen oxides (NOx) rise when brake thermal efficiency falls [Paswan] [2]. The simulated findings and experimental values were contrasted. Better outcomes were obtained using the LPO20 (lemon peel oil) and 10 % DEE (diethyl ether) combination. In comparison to test results using LPO20 10 % DEE, Diesel RK software demonstrated improved results for BTE, NOx, and cylinder pressure [Raju, V. D] [3].

Using diesel, microalgae biodiesel (MAB), binary, and ternary MAB fuel blends, exhaust gas emissions of a single-cylinder, compression-ignition engine were studied for various loads and compression ratios, and the experimental findings were compared with diesel-RK software. As compared to diesel, MAB mix reduces smoke, NOx, particulate matter, and exhaust gas temperature [Rajak, U] [4]. Spirulina microalgae, diethyl ether (DEE), n-butanol (nB), and 95 percent diesel fuel with 5 percent hydrogen were all used in the experiment (SMA). Using software called Diesel-RK Model, the fuels were assessed in a single-cylinder CI engine. According to the experimental findings, a 5%H95%DEE mix resulted in the maximum specific fuel consumption [Chaurasiya,] [5].

Tested and compared to normal gasoline were four separate mixes (20 %, 5 % each) made from different energy sources such spirulina, soybean oil, waste oil, and jatropha. Test results were assessed using the Diesel-RK program. Particulate matter, cylinder pressure, and brake thermal efficiency all dropped by roughly 4.18 %, 6.86 %, and 6.4 %, respectively. The test results were compared with the diesel-RK program under the identical operating parameters [Srivastava,] [6]. Lowered NOx and greater CO and HC emissions were the results of employing Ternary fuel (diesel, biodiesel, and ethanol) in a TRCC (Toroidal Re-entrant Combustion Chamber; [Venu, H] [7]. Using the program Diesel-RK, the numerical analysis of the combustion, performance, and emission characteristics under various loads and compression ratios including 16.5, 17.5, and 18.5 was conducted. In the testing, fuels including Jatropha mix, Rapseed, and Jojoba B20 blends were employed. By utilizing a B20 blend instead of diesel fuel, cylinder temperatures were found to be 8.2 % lower for Jatropha curcas, 13.47 % lower for rapeseed, 63.85 % lower for jojoba oil, and 42.2 % lower for NOX emissions [Rajak, U., Nashine] [8].

Many publications claimed that a few greenhouse gases have been significantly reduced. According to Vezir Ayhan et al. [9], the emission characteristics of SFME with B10, B20, and B50 revealed a 5.25 % decrease in carbon dioxide emissions. The results of employing biofuel blends in diesel engines (CRDI) operating at 2000 rpm under various load circumstances yielded an increase in NOX content of 7.3%–12.3 % and a decrease in CO of 12.2%–45.1 %. WCO biodiesel is utilized in single-cylinder, four-stroke diesel engines with a 2800 rpm operating speed. According to the findings, carbon monoxide was reduced by 65.4 % and exhaust gas temperatures dropped by 1.4 % [LlkerOrs et al., 2011] [10].

There is an attempt made to reduce diesel engine emissions. Due to a rise in unsaturation and the complexity of the fuel's molecules, the biodiesel blends were analyzed, and the findings revealed higher amounts of nitrogen oxide (NOX) [Labeckas G. et al.] [11]. The results of testing biofuel blends at low and ambient temperatures revealed high cloud point at −70C and substantial HC emissions. Due to its high oxygen capacity, it also demonstrated decreased particle emissions under the two conditions given [Calle Asensio et al.] [12]. Biodiesel (RME) was employed in the CI engine experiments at a 3000 rpm engine speed. Because of the high combustion temperature and high Cetane value, it demonstrated a 13 % reduction in THC. Moreover, it demonstrated a 22 % decrease in carbon monoxide [Kamil Duda et al.] [13]. A decreased thermal efficiency of 5.88 % was discovered in biodiesel B20 produced from Cymbopogonflexuosus [Balasubramanian. et al.] [14]. According to Maciej Mikulski et al. [15], the ACDR engine running on biodiesel at 3000 rpm exhibited a 3.2%–13.8 % increase in fuel and a 1.6%–7.8 % loss in brake thermal efficiency.

Liquid fuels are ideal for internal combustion engines because they are simple to handle, store, and transport and have a high calorific value. The BSFC and BTE of the PO20 BHT mix are greater than diesel fuel by 11.4 % and 5.1 %, respectively. Compared to diesel fuel, CO emissions from the PO20-butanol mixture are 37.5 % lower, but NOx emissions are 1.9 % higher [Prabu, S. Senthur et al.] [16]. According to Asokan, M. A. et al. [17], Juliflora biodiesel B100 had a BTE of 31.11 % at maximum power, which was very similar to diesel (32.05 %). Although lowering smoke, NOx, and CO emissions in comparison to diesel by 32.4 percent, 27.6 percent, and 41.76 percent, respectively, using ternary blends of jatropha biodiesel, pentanol, and diesel in a diesel engine resulted to a slight drop in BTE [Appavu, Prabhu et al.] [18]. the enrichment of BSFC and BTE in diesel engines running on blends of decanol and neem biodiesel. UHC, smoke, and CO concentrations all decreased, although there was a little increase in NOx concentrations [Devarajan, Yuvarajan et al.] [19].

Investigations and research are continually being conducted to identify low-cost techniques to reduce emissions from diesel engines as environmental problems become more important. performed analysis on the bio-diesel blends on “Kirloskar CI diesel engine” at 1200 rpm. They saw an increase in blends' thermal efficiency of roughly 6.2 %. This was brought on by the bio-decreased diesel's frictional drag. When alcohol is used, the NOX tends to decrease due to its cooling impact. Also, they noted a pattern of decreasing fuel costs with more alcohol [Rajesh et al.] [20].

CI engines used in the transportation sector are one of the primary causes of air pollution. According to Drenth et al. [21], there was an observation of a 5 % improvement in braking efficiency as a result of the biofuel's improved lubricating properties. owing to incomplete combustion, carbon dioxide emissions from small-to medium-sized loads have increased by roughly 7 %. Nitrogen oxide emissions were around 3.5 % lower while the engine was running at no load than when it was operating at peak loads, according to Ndayishimiye et al. [22].

Biodiesel (B100) demonstrated thermal efficiency of 32.4 % in an experimental study, compared to diesel's 29.98 % for the identical load test. With increased load and lowering speed, the exhaust soot value dropped from 3.7 to 6.7 [23]. Likhanov et al. B20D80 exhibited the highest example of thermal efficiency for all mixes with a change in compression ratio up to 19. The engine used, its operating circumstances, the additives that change a few features, and the chemical species employed all have a significant role on the emission and performance of biofuel created.

According to the experiment findings, adding mandarin essential oil to diesel fuel causes a 2.5 and 3 % reduction in HRR and cylinder pressure, respectively. As compared to the basic fuel, the MO10 and MO20 combinations' NOx intensity rose by 45 % and 25 %, respectively, but their smoke opacity, UHC, and CO levels decreased by 27 %, 44 %, 20 %, and 17 %, respectively. For the MO20 and MO10 mixes, the SFC declines by 22 % and 5 %, respectively [Gad, M. S. et al.] [24]. When the heating value of the blended fuels reduced, the maximum pressure fell as MEWCO mixes were raised. In return for lower carbon emissions, they promised to decrease nitrogen oxides (NOx) [Al-Dawody, M. F., A. A. Jazie, and H. A. Abbas] [25].

Algae's fast growth rates, controllable growth densities, and high oil yields often prompt significant investment in algae-to-biofuel conversion. However, there are several challenges to making algae a cost-effective alternative to petroleum, thereby reducing CO_2_ emissions. These challenges encompass optimizing cultivation methods and locations, improving oil extraction techniques, and refining fuel processing [Mariadhas A. et al.] [26].

The experimental study evaluated the effects of RCCI (reactivity-controlled compression ignition) on the performance, emissions, and combustion characteristics of a CRDI engine. A fuel blend consisting of 20 % biodiesel, 80 % diesel, and a NaOH catalyst was used. The engine investigation involved three different injection strategies: 10 % penetration RCCI, 20 % penetration RCCI, and 30 % penetration RCCI. As the charge increases, there is an increase in CO2, hydrocarbon emissions, and smoke opacity values [Anish, M. et al.] [27].

According to the investigations, Zinc oxide nanoparticles were combined with tungsto-phosphoric acid (TPA) to serve as a catalyst for converting waste cooking oil into biodiesel. A mixture of 10 wt% zinc oxide nanoparticles and 90 wt% TPA was employed. For engine testing and analysis, the synthesized biodiesel was used as blends of B10, B20, and B100. The biodiesel blends exhibited lower carbon emissions compared to conventional diesel, which had significantly higher CO and HC emissions. However, the biodiesel blends produced higher NOx emissions than diesel [Jayaraman, J. et al.] [28].

The study examines the impact of biosynthesized zirconium nanoparticles in diesel fuel, focusing on combustion, emissions, and engine performance. The results show that adding 20 nm zirconia nanoparticles to pure diesel increased thermal efficiency by 4.9 %, reduced specific fuel consumption by 2.9 %, and reduced diesel smoke, hydrocarbon, CO, and NOx emissions by 13 %, 20 %, 25 %, and 29 %, respectively [Anish M et al.] [29].

This research examined the combustion, efficiency, and emission properties of a single-cylinder diesel engine using nanoparticles. The ultrasonicator, silicon carbide, and carbon nanotubes increased mixing and chemical reactivity, improving engine performance. The nanoparticle-based engine had 20 % higher braking thermal efficiency and reduced nitrogen oxide, carbon monoxide, hydrocarbon, and smoke emissions [Joy N et al.] [30].

Utilizing Saffron Seed Oil in Internal Combustion Engines (ICE) as a substitute for biodiesel has favorable environmental impacts of reducing greenhouse gas and exhaust emissions. However, it is crucial to manage the cultivation and production processes sustainably to mitigate potential negative impacts such as deforestation, water pollution, and competition with food crops.

The novelty of this study is a thorough investigation of American saffron oil as a prospective feedstock for biodiesel production, accompanied by a meticulous examination of experimental and computational methods to assess the efficiency and environmental advantages of ASOME-diesel blends. The results of this research have the potential to facilitate the commercialization and extensive use of American saffron oil as a feasible and environmentally friendly substitute for conventional diesel fuel.

The main objective of this work is to provide insights into the potential of American saffron oil as a sustainable alternative to conventional diesel, focusing on its efficiency and environmental benefits. Compare the experimental data and simulation results to assess the engine performance and emissions reduction potential of the ASOME-based biodiesel blends.

## 2 Materials and methodology

### 2.1 Oil extraction

**Fig. 1:**
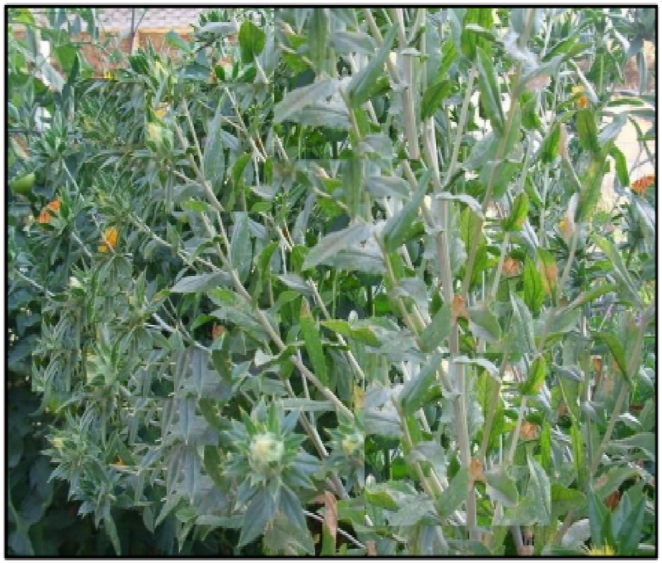
American Saffron plant.

**Fig. 2:**
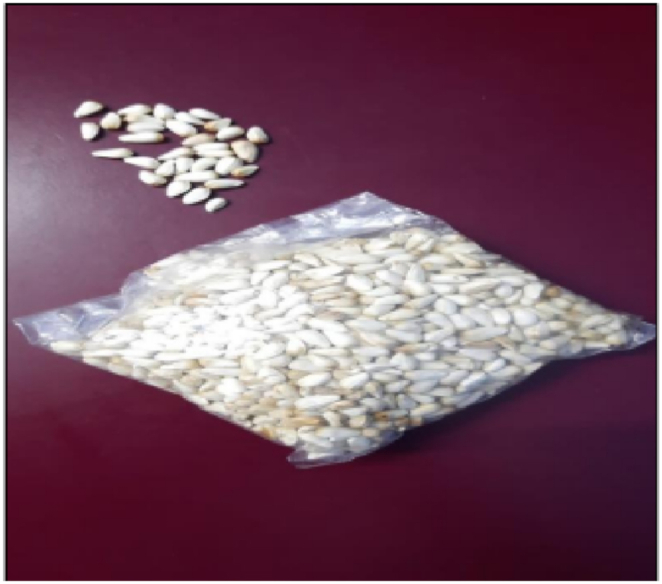
Saffron seeds.

**Fig. 3:**
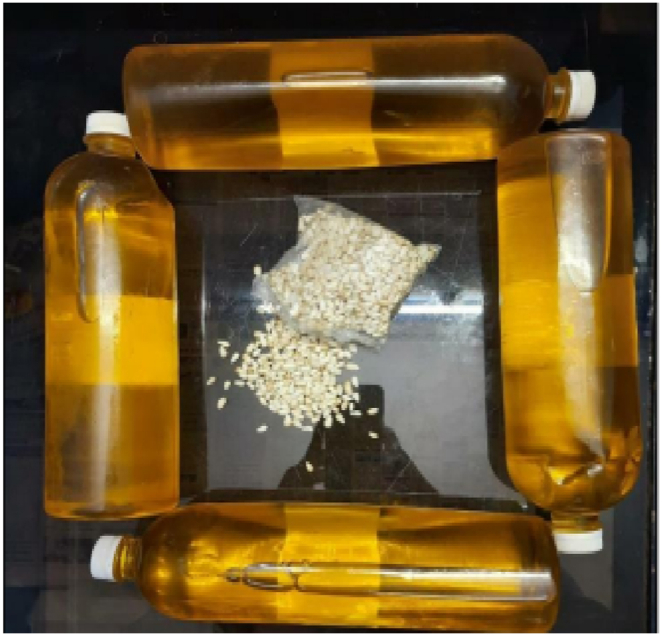
Saffron oil.

**Fig. 4:**
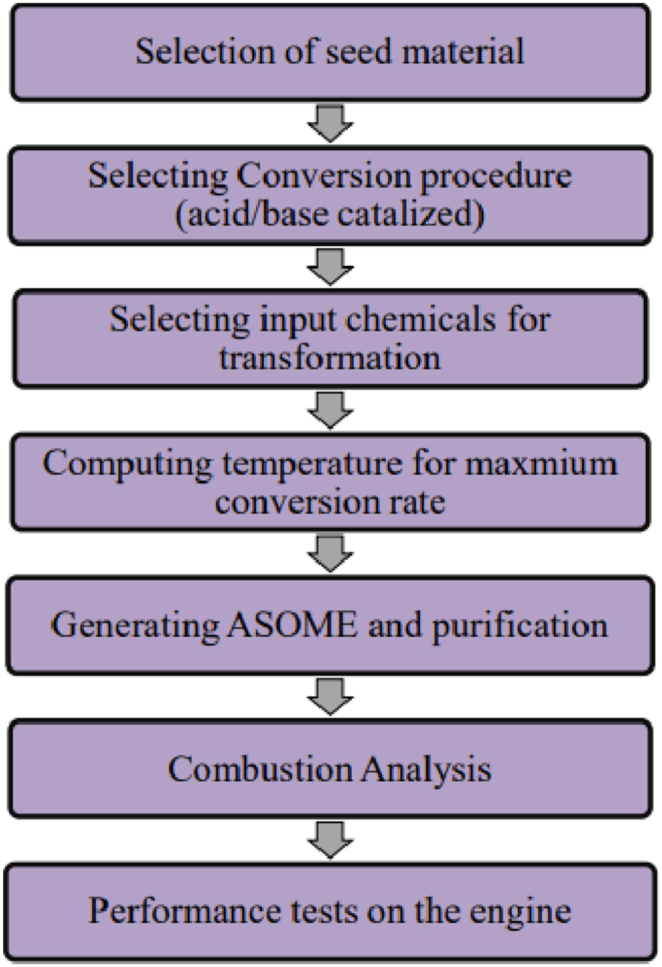
Process Flow chart.

The American saffron seeds undergo preliminary processing, which includes the removal of soft, minute fibers, husk, mud, and other extraneous materials. The prepared seeds are conditioned after that. The process of conditioning involves heating the seeds to a high temperature (approximately 800 C) Chouaibi and L. Rezig [31]. In seed cells, the oil is found as triacyl-glycerol. After oil extraction, it must go through a number of steps. One may refer to these steps as post-processing activities. When the liquid-filled membranes are being broken down and evacuated, the oil is being extracted by grinding. Fatty acids are the primary oil component required for activity. Membrane that contains oil ruptures during extraction. Not only the oil vessels but also the other vitamins in the liquid phase, the glycerophospholipids that make up the membrane, and the sterols will be released during this process. Some of these may not be affecting the result at all. Yet, additional factors might impact the ultimate crop and its quality. In addition to degumming this, boiling it to roughly 60 °C is done to remove the water. Fig. 1, Fig. 2, Fig. 3 depict the plant, seeds, and oil of the American Saffron. Fig. 4 depicts the process flow diagram.

### 2.2 Bio-Diesel production

The methyl ester that has been synthesized is then first heated to around 60 °C. A reaction beaker is drawn with around 125 gm of oil (750 ml). Methanol or carbinol is added at a ratio of 4:1 by weight to oil in the reaction mixture. Because of the differences in their surface tension qualities, a high temperature must be maintained to change (reduce) it. Afterwards the stirrer helps to create a perfect emulsion, increasing the number of reaction sites. According to Omkaresh, B. R., and Suresh, R. [32], it is kept at 1000 rpm.

According to the chemical rate modeling findings, which indicated an optimal range of [55^0^C–59 °C], 57 °C is chosen as the working input. Hydrogen sulfate- 0.4w/w% (50 gm) is used to complete the first stage of the reaction for the specified period of time (35 min).

Esterification is a chemical reaction that involves the conversion of carboxylic acids to esters by the reaction with alcohols, in the presence of an acid catalyst. Oleic acid is a monounsaturated omega-9 fatty acid that can undergo esterification to form esters, which are commonly used in the production of biodiesel, cosmetics, and other industrial applications.

**Figure:**
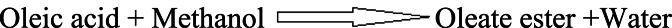

$$\text{RCOOR}’+R”\text{OH}\;\overset{NaOH,\Delta }{\Leftrightarrow }\;\text{RCOOR}”+R’\text{OH}$$

The above base catalysed after certain progression of acid catalysed reaction is started. This is so as to increase conversion efficiency.

In esterification, the carboxylic acid reacts with the alcohol to form an ester and water. The acid catalyst facilitates the reaction by protonating the carboxylic acid, making it more reactive towards the alcohol.

In transesterification, the triglyceride reacts with an alcohol in the presence of a catalyst (either acid or base) to produce fatty acid esters and glycerol. The esterification and transesterification reactions are similar, but the starting materials are different. In transesterification, the starting material is a triglyceride molecule, whereas in esterification, the starting material is a carboxylic acid.

$$\text{RCOOR}’+\text{NaOH}\;\overset{H_{2}O}{\to }\;\text{ROON}\;a^{+}+R’\text{OH}\;-------\;\left(\text{Saponification}\;\text{reaction}\right)$$

The next catalyst is NaOH (0.6w/w%; 75 gm). The start of 99 % response must occur within a certain window of time, or 132 min. Titration is what determines how far along it is [Ulakpa, W. C., Ulakpa] [33].

**Fig. 5:**
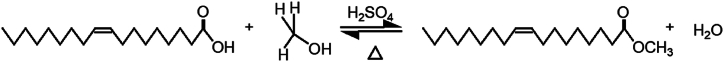
Esterification of oleic acid (one of major component).

**Fig. 6:**
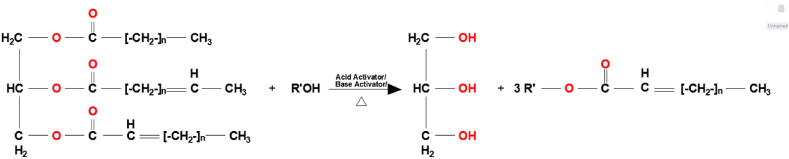
Net reaction of esterification/transesterification.

The mixture is allowed to settle for 24 h in a separating funnel after the 138 min reaction stage. ASOME (American Saffron Oil Methyl Ester), glycerin, ASOME-.Na+, CH3OH, and NaOH are present in the final products. When some settling time has passed, the biodiesel will be separated from the glycerin that has a greater specific weight than ASOME. Any polarization already present will be negated by the addition of hydrogen sulfate, allowing the base to be converted. By vigorously mixing with the addition of water, the soap ASOME-.Na+ and any salt will be removed [Takase, M., Bryant] [34].The pictorial form of reactions is illustrated in Fig. 5, Fig. 6.

**Fig. 7:**
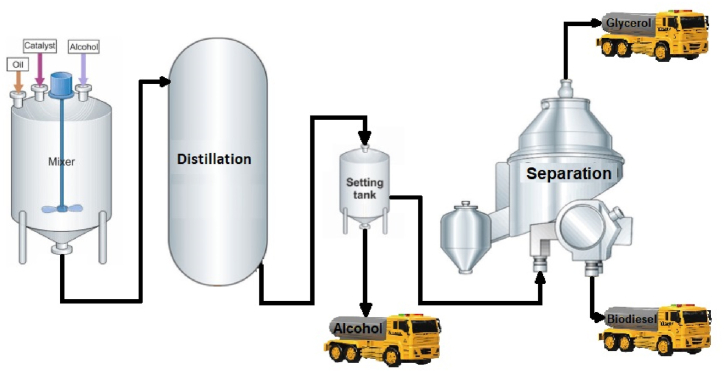
Biodiesel production process.

The mixture is allowed to settle for 2 h for water de-emulsification from ASOME. It will be heated about 80 °C for the conversion of water into vapour and the remaining methanol (boiling point of CH_3_OH- 64.7 °C) will be distilled over the top. Fig. 7 Represents the biodiesel production process.

### 2.3 Combustion in CHEMKIN

**Fig. 8:**
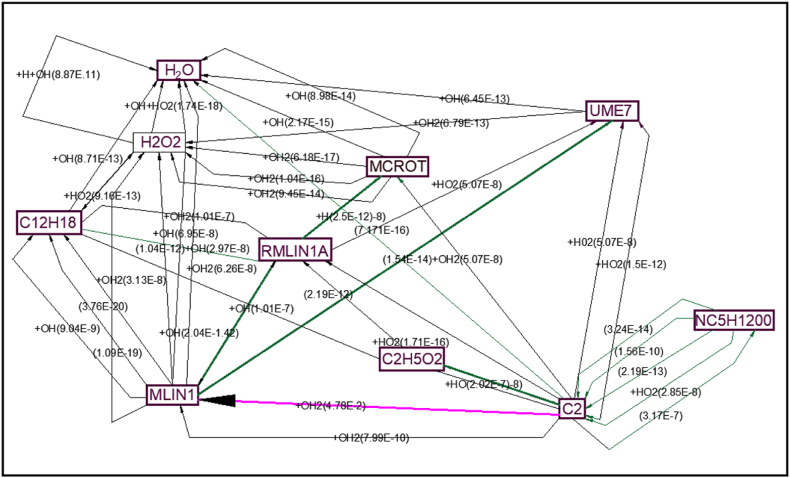
Combustion flow process.

The data on the combustion of linoleic acid methyl ester and oleic acid methyl ester is derived from the literature since the oil has a large quantity of linoleic and oleic acids [Hernández, M. L., Sicardo] [35]. The necessary data is submitted for the combustion analysis. The repetitive responses have been eliminated and the zeldovich mechanism added to these files. The "JANAF thermodynamic tables" are used to retrieve the thermodynamic coefficients' missing data. The values are sent to the program as input. With the other settings set to default values, the aforementioned simulation is performed at various equivalence ratios to forecast the output flame temperature and the burning flame's laminar velocity [Zehni, A., & Saray, R. K] [36]. The combustion flow process is shown in Fig. 8.

### 2.4 Fuel testing

The four fuels utilized in this experimentation are prepared as methyl esters (ASOME) blends- B20D80, B40D60, B60D40 and the other fuel is regular diesel. The fuel properties are shown in Table 1. The free fatty acid data and gas analyser uncertainty analysis are shown in Table-2, Table-3.

**Table-1:** Fuel properties.

Property	ASOME			D100	Test Method
	B20- D80	B40-D60	B60-D40		
Flash Point (^0^C)	62	74	80	68	ASTMD93
Gross Calorific Value (Kcal/kg)	10095	10000	9534	10205	IS 1448 (p-6)
Cloud Point (^0^C)	−12	−9	−9	3	IS 1448 (P-10/Sec-1)
Pour Point (^0^C)	−15	−12	−12	−3	
Cetane Number	48.68	49.37	51.3	48	ASTMD613
Density (kg/m^3^)	841	852	885	830	ASTMD792

**Table-2:** Fatty acid data.

Time (minutes)	FFA (weight)
12	14.87 gm
28	7.07 gm
54	3.81 gm
106	1.988 gm

**Table-3:** Gas analyser uncertainty analysis.

Parameters	Instrument uncertainty
CO(%)	±0.025
CO_2_(%)	±0.025
NO_x_(ppm)	±8
HC(ppm)	±9

**Fig. 9:**
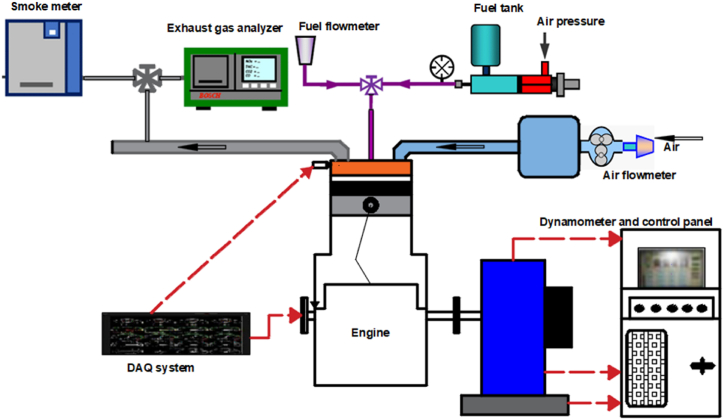
Engine lay out.

**Fig. 10:**
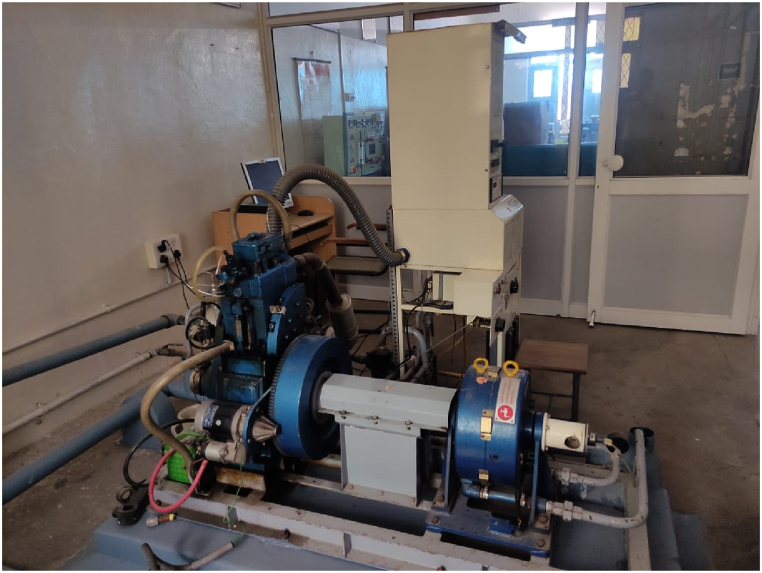
Experimental engine set-up.

The investigations were conducted using the experimental setup and Kirloskar single cylinder 4-stroke direct injection water cooled diesel engine layout shown in Fig. 9, Fig. 10. For load variation, an eddy current dynamometer was used. The engine setup comprises of an IC engine soft.v9-equipped computer system and a data gathering system (DAS). Via an ECU, a diesel engine's DAS was utilized to transfer data about engine operating parameters to computer software (Engine control unit). In response to input from the user, the engine software changes the different operational parameters of the engine. A speed controller was used to change the engine's speed, while a load controller was used to change the load. the air chamber from which the combustion chamber receives its air supply. The gasoline burette was used to measure the engine's fuel consumption. The engine panel board is where this burette is mounted. Before starting the engine verify the wiring connections of all electrical and electronic components. On the dynamometer, a load cell was installed to detect changes in the load and transmit that signal to the program through a data gathering system. To gauge the engine's exhaust gas temperature, thermocouples were built within the engine. For the purpose of measuring engine exhaust emissions such CO, NO_X_, CO_2_, HC, and smoke opacity, the gas analyzer was attached to the engine exhaust system. The engine data is sent to the "Diesel RK" program. Table-4 lists the engine specifications.

**Table-4:** Engine specifications.

Parameters	Specifications
Test engine	Research engine setup, 4 stroke water cooled engine
Piston shape	cylindrical
Piston dimensions	87.5 mm–87.6 mm
Make	Kirloskar
Number of cylinders	1
Rated Speed and Rated Power	1500 rpm and 3.5 kW
Stroke Length	110 mm
CR Range	12:1 to 18:1
Orifice diameter	20 mm
Dynamometer arm length	185 mm, Eddy current Dynamometer
Injection point variation	0 to 25^o^ BTDC
Type of starting	Key or Crank start

In engine testing, various parameters such as load, fuel consumption rate are measured and calculated to evaluate the engine's efficiency, power output, and emissions. The following relations are used to calculate the final data.

#### 2.4.1 Experimental testing

Brake Thermal efficiency:

$$\text{BTE}\left(\%\right)=\frac{\text{Brake}\;\text{Power}}{\text{Fuel}\;\text{Power}\;\text{Input}}\;x\;100$$

Specific Fuel consumption:

$$\text{SFC}\;\left(g/\text{kWh}\right)=\frac{\text{Brake}\;\text{Power}\left(g/h\right)}{\text{Fuel}\;\text{Consumption}\left(\text{kW}\right)\;}\;x\;1000$$

#### 2.4.2 Emissions

$$\text{Reduction}\;\left(\%\right)=\frac{\text{Emission}\;\text{in}\;\text{diesel}-\text{Emission}\;\text{in}\;\text{biodiesel}\;\text{blend}}{\text{Emission}\;\text{in}\;\text{diesel}}\;x\;100$$

The uncertainty analysis of engine is shown in Table 5.

**Table-5:** Uncertainty Analysis of engine parameters.

Parameters	Uncertainty
Speed(rpm)	±1
Time(s)	±0.1
Load(N)	±0.2
Temperature(^o^C)	±1
Brake Power(kW)	±0.5
Brake Thermal Efficiency	±0.6
Specific fuel consumption	±0.5
Torque	±0.5
Pressure(bar)	±1

## 3 Results and discussion

### 3.1 Combustion analysis

**Fig. 11:**
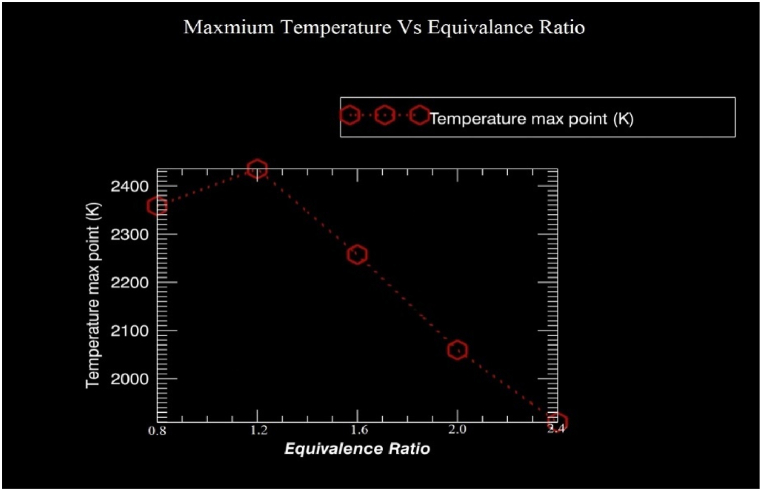
CFT_max_(K) vs Equivalence ratio(Φ).

As shown in Fig. 11, the equivalency ratio has a direct impact on the maximum temperature. Due to the computer's Memory limitations, the grid size has been set to low values (size = 300) (exceeding the maximum set space). The range has 5 division points and ranges from 0.8 to 2.4. As can be observed, the temperature (maximum) is lower owing to a slower reaction development in lean mixtures than in oxygen. Equivalence ratio (1.2), or around 2395 K, is where the highest combustion flame temperature is found. Further raising the causes, the mixture to become rich, which indicates less air in the mixture. The CFT is dropped because the mixture won't completely burn since the air percentage is lower.

**Fig. 12:**
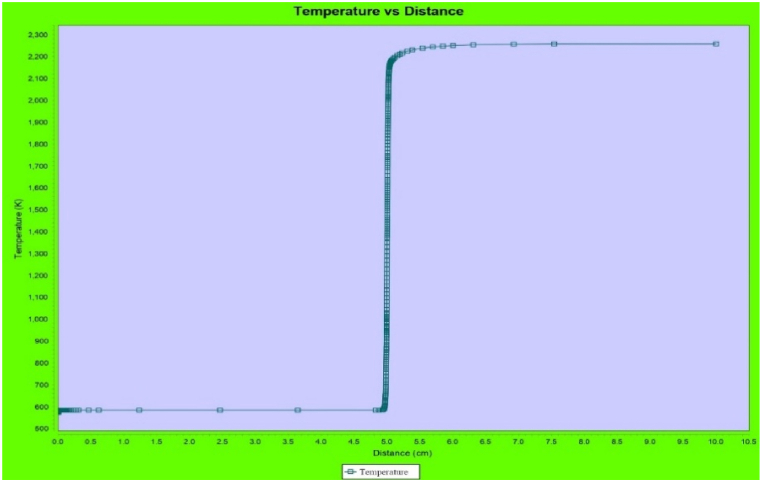
CFT (K) vs Distance in Chamber (cm).

Fig. 12 shows the territories in the combustion at Φ = 1.2. A 1000 grid point density is assumed. The pre-heating area reaches x = 0 cm–4.8 cm in length. The combustion zone is between 4.8 and 5.5 cm in diameter. The most prolific stage of combustion takes place in this area. Around 92 % of the heat produced at the stage's conclusion. Beyond x = 5.5 cm, a sluggish, steady temperature gradient is seen, and it takes time for it to stabilize. At x = 10 cm, the highest temperature, or 2296 K, is shown. At 10 cm, the temperature stabilizes. This area is known as the "product zone.".

### 3.2 Efficiency analysis

**Fig. 13:**
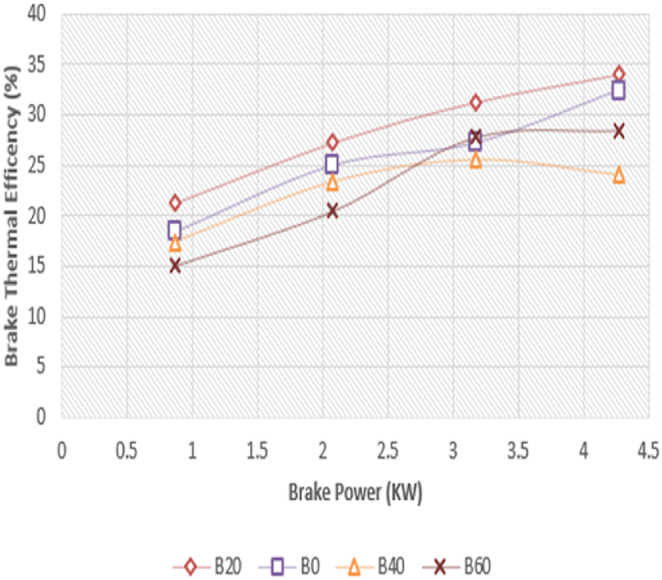
Computed- BTE (%) vs BP (KW).

**Fig. 14:**
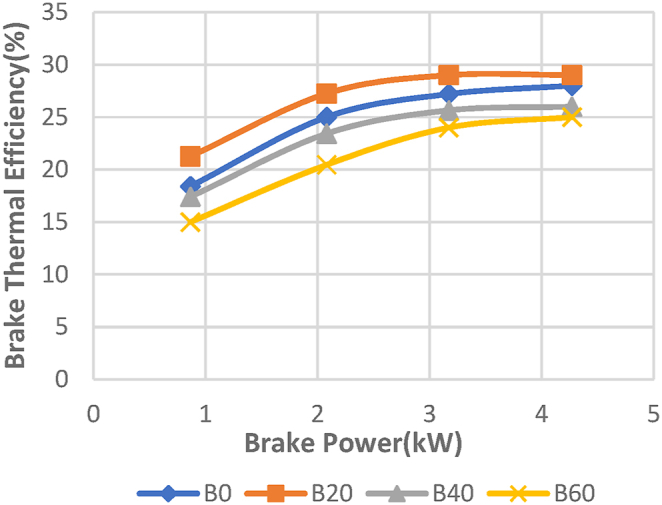
Experimental- BTE (%) vs BP (KW).

The fluctuation of braking power with brake thermal efficiency (BTE) of the Diesel-RK simulation is shown in Fig. 13. Brake thermal efficiency, which measures the percentage of fuel energy converted to useable work, is one of the most crucial engine performance metrics [Paul G et al.] [37]. The experimental data' braking thermal efficiency is shown in Fig. 14. With diesel-RK, the ASOME B20 brake thermal efficiency is high and is around 13.7 % higher than experimental data at maximum load. Efficiency figures for the B20 blend at maximum load conditions are 33.1 % for simulation and 29 % for experimental measurements. Under peak load conditions, the remaining values for Diesel-RK are 32 %, 24.12 %, and 28 % for B0, B40, and B60, respectively. For B0, B40, and B60, the experimental values are 28 %, 25.1 %, and 24.8 %, respectively. When the load grows, the BTE rises. This is because the heat generated by the combustion within the cylinder has increased.

### 3.3 Fuel consumption

**Fig. 15:**
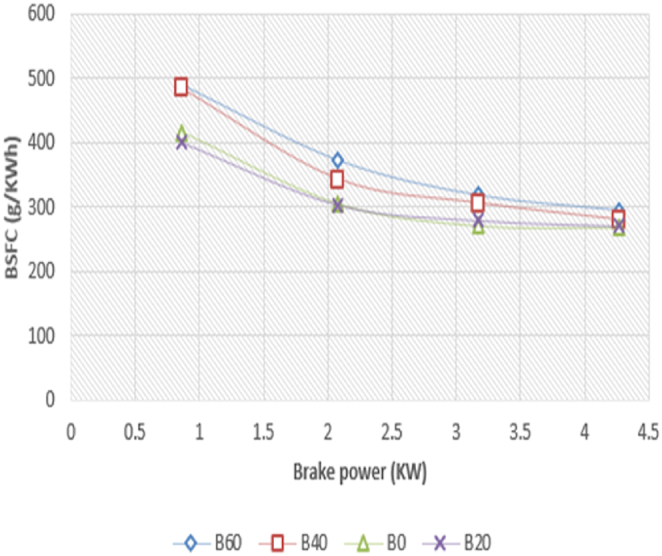
Computed- BSFC (g/KWh) vs BP (KW).

**Fig. 16:**
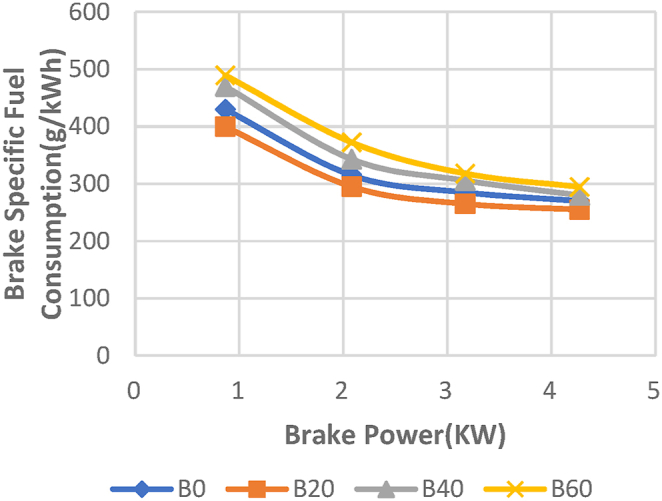
Experimental- BSFC (g/KWh) vs BP (KW).

The fluctuation of "BSFC" in relation to Brake power for Diesel RK simulation of ASOME mixes and diesel is shown in Fig. 15. When evaluating an engine's performance, one significant aspect to take into account is the brake specific fuel consumption (BSFC). The BSFC is shown to fall with increasing engine load for all test fuels. This is made feasible by the cylinder's more effective combustion under greater load situations [38].[Kumar Kadian]. The experimental depiction of a particular fuel consumption with braking power is shown in Fig. 16. With diesel-RK, the BSFC of ASOME B20 is high and is about 5.7 % higher than experimental data at maximum load. The BSFC values for B20 blend are 255 g/kWh for experimental findings and 270.74 g/kWh for simulation at maximum load conditions. The remaining Diesel-RK values under peak load conditions are 268.9 g/kWh, 276 g/kWh, and 298 g/kWh for B0, B40, and B60, respectively. The experimental values for B0, B40, and B60 are 270 g/kWh, 279.7 g/kWh, and 294.5 g/kWh, respectively. At greater load circumstances, the modeling and experimental findings with ASOME blends and diesel are more similar.

### 3.4 Emission analysis

**Fig. 17:**
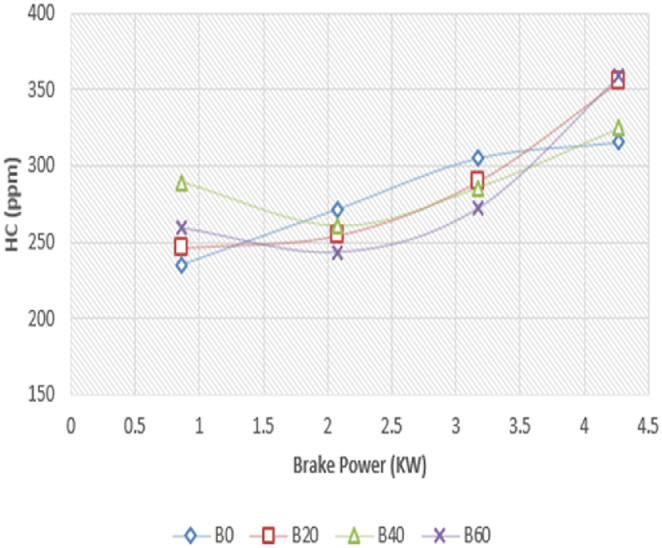
Computed- HC (ppm) vs BP (KW).

**Fig. 18:**
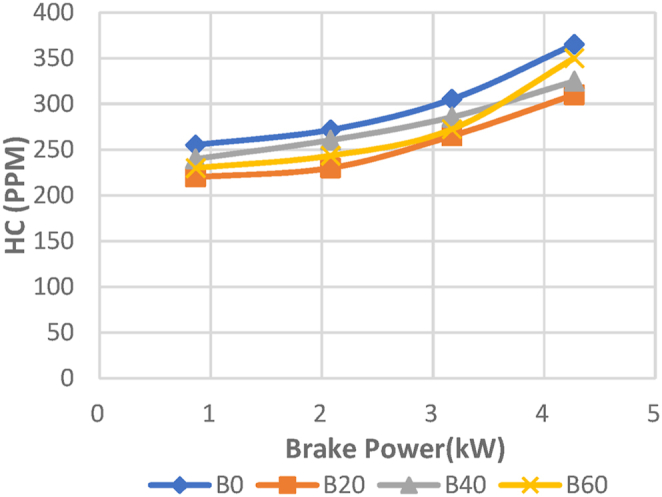
Experimental- HC (ppm) vs BP (KW).

In the Diesel-RK simulation of ASOME mixes and diesel, Fig. 17 shows how hydrocarbon emission (HC) varies with braking power. Since the fuel and air used in the CI engine are heterogeneous, there is incomplete fuel combustion and the development of HC emissions [Roy, A., Dabhi, Y] [39]. UHC emissions decrease with increasing engine load for all test fuels. The experimental depiction of HC with braking power is shown in Fig. 18. The ASOME B20's HC emissions are low for diesel-RK and are just around 2.59 % higher than experimental results under full load conditions. For blend mixes of B0, B40, and B60, the simulated values of diesel RK are determined to be 315.5 ppm, 324.7 ppm, and 359.22 ppm, respectively. The experimental values for B0, B40, and B60 are 365 ppm, 324.71 ppm, and 350 ppm, respectively. At peak loads, the engine operates under high temperature and pressure conditions. Under these conditions, there may not be enough oxygen available in the combustion chamber to fully oxidize all the hydrocarbons, leading to incomplete combustion and increased HC emissions.

**Fig. 19:**
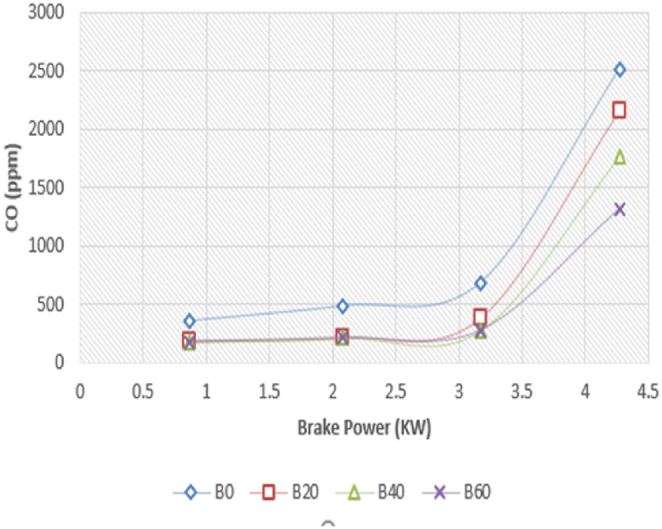
Computed- CO (ppm) vs BP (KW).

**Fig. 20:**
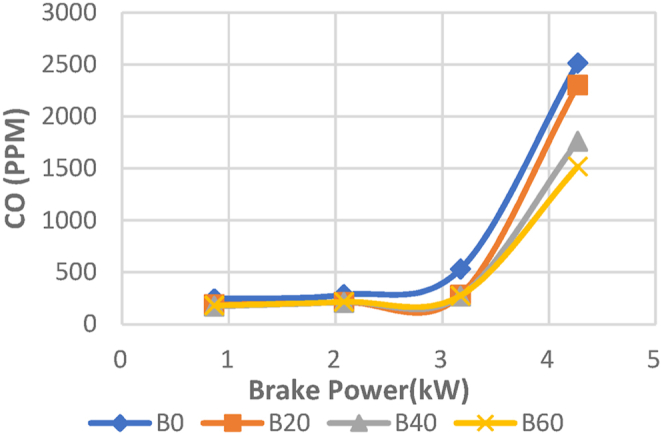
Experimental- CO (ppm) vs BP (KW).

For the Diesel RK simulation of ASOME mixes and diesel, Fig. 19 displays the fluctuation of carbon monoxide (CO) with braking power. Uncompleted combustion results in the production of carbon monoxide (CO), a colorless, odorless, and flavorless gas that is usually regarded as a health threat [40]. Elnajjar, E.; Al-Omari. The experimental study of CO emissions is shown in Fig. 20. No load to part load conditions result in the same CO emissions. The CO emissions then dramatically rise. ASOME B60 has low CO emissions for diesel RK and is 14.3 % less than trial findings. For B0, B20, B40, and B60, respectively, the simulated values for diesel RK are determined to be 2500 ppm, 2200 ppm, 1750 ppm, and 1300 ppm. The experimental values for B0, B20, B40, and B60 are 2512 ppm, 2300 ppm, 1760 ppm, and 1517 ppm, respectively. When the mix proportion in the diesel increases, the CO emissions drop. Additionally, the concentration of CO emissions rises mostly at high loads as a result of excessive fuel injection and insufficient oxygen for full fuel burning. At high load conditions, the fuel may not be atomized properly due to the increased fuel flow rates, leading to larger fuel droplets. These larger droplets can result in poor mixing with the air and incomplete combustion, leading to increased CO emissions.

**Fig. 21:**
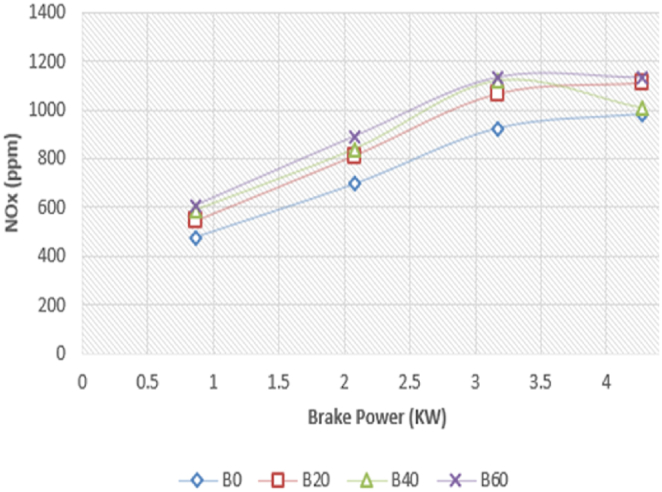
Computed- NO_X_ (ppm) vs BP (KW).

**Fig. 22:**
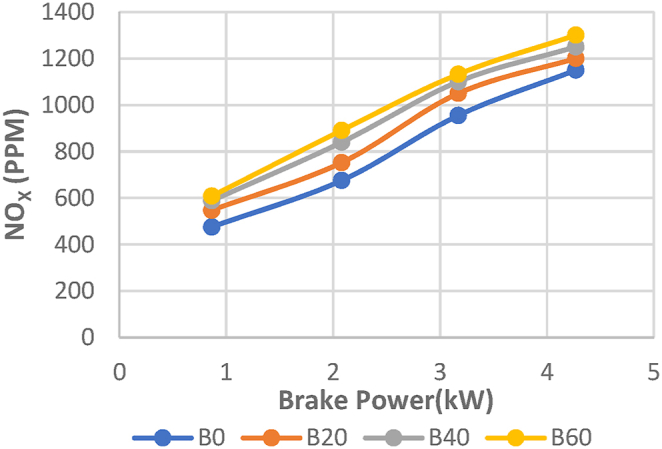
Experimental- NO_X_ (ppm) vs BP (KW).

The NOx emissions with braking power for the Diesel RK simulation with ASOME mixes and diesel are shown in Fig. 21. For all of the tested fuels, NOx emissions rose when braking power was increased. There are 975 ppm, 1120 ppm, 1000 ppm, and 1150 ppm of NOx emissions for B0, B20, B40, and B60, respectively. The experimental results for diesel and ASOME mixes are shown in Fig. 22. From no load to full load, there is a rise in nitrogen oxide emissions. When a diesel engine is working at low loads, nitrogen oxide emissions are lower; when the engine is operating at full loads, they are greater. The nitrogen oxides produced during the combustion of biodiesel blends rise more slowly than those of pure base fuel due to the increased oxygen availability in the fuel [P. V. Emmalai et al.]. [41]. B0, B20, B40, and B60 are found to have experimental values of 983 ppm, 1029 ppm, 1028 ppm, and 1130 ppm, respectively. As compared to other ASOME mixes, the NOx emissions for B60 experimental results are higher.

**Fig. 23:**
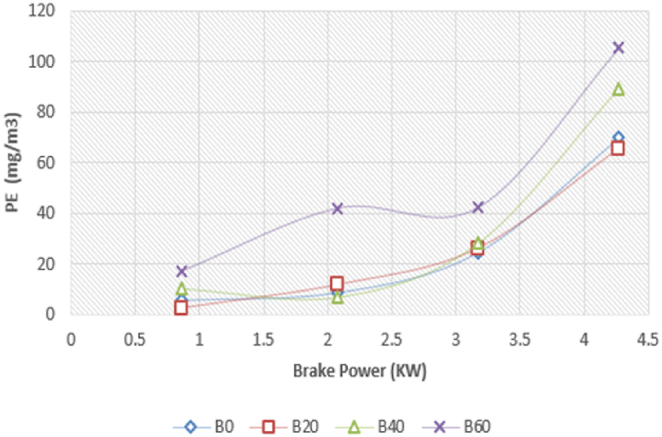
Computed- PE (mg/m^3^) vs BP (KW).

**Fig. 24:**
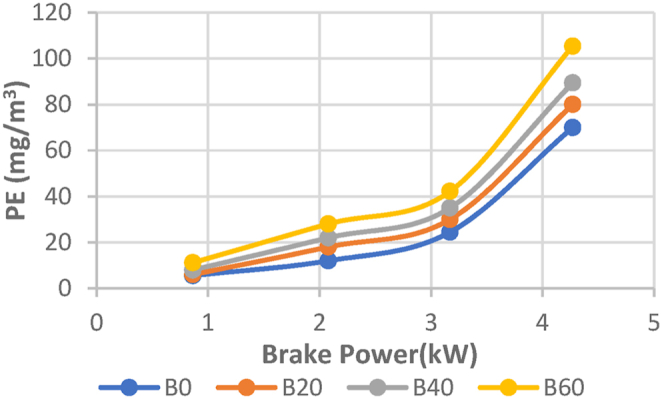
Experimental- PE (mg/m^3^) vs BP (KW).

The particulate emission with brake power for the Diesel RK simulation with ASOME mixes and diesel is shown in Fig. 23. For the diesel-RK simulation, the particulate emissions for B0, B20, B40, and B60 are 70 mg/m3, 62 mg/m3, 89 mg/m3, and 108 mg/m3, respectively. For experimental data, Fig. 24 depicts the fluctuation of particulateemission with brake power. According to experimental data, the particulateemissions for B0, B20, B40, and B60 are 69 mg/m3, 70 mg/m3, 91 mg/m3, and 105 mg/m3, respectively. B60 particulate emissions are more compared to diesel fuel. Biodiesel has a higher oxygen content than conventional diesel, which can sometimes lead to incomplete combustion and the formation of soot particles. Karin P. et al., 2022 [42].

## 4 Conclusion

The influence of various engine parameters and load factors on dual engine performance and emissions has been studied. Single cylinder diesel engine is used in this study and operated with biodiesel blends extracted from American Saffron seed oil. The following are the major findings of the study.

• This work found that producing biodiesel from American Saffron seed oil using a one-step transesterification method is feasible and it can be used in existing IC engine.
• The combustion zone is between 4.8 and 5.5 cm in diameter. The prolific combustion area is observed to be takes place in this area.
• It is found that the blendB20 produces highest brake thermal efficiency at maximum load condition. The experiments showed that the efficiency increases by 29 %.
• The brake specific fuel consumption of ASOME B20 is high for diesel-RK and is about 5.7 % compared to experimental data at maximum load.
• The HC and CO emissions of ASOME B20 are low for diesel-RK and is about 2.59 % and 14.3 % respectively.
• The NO_x_emissions showed similar trend in diesel-RK. The NO_x_ emissions for B60 experimental data is more compared to other ASOME blends.
• All the test results of ASOME are compared with diesel fuel. Further, the experimental data is compared with simulation data (RK-Diesel).

## Future recommendation

Based on the findings of the study on American Saffron Oil methyl ester (ASOME) as a potential feedstock for biodiesel production and its impact on diesel engine performance and emissions. Further research may focus on optimizing the blend ratios of ASOME with diesel to achieve better performance and emissions. Further the investigations can extend with various fuel injection pressures and injection timings.

**Table:** NOMENCLATURE:

ASO	American Saffron Oil
ASOME	American Saffron Oil Methyl Ester
BSFC	Break Specific Fuel Consumption
CFT	Combustion Flame Temperature
COL	CarthamusOxyacanthusL.
ERM	Endo-Plasmic Reticulum Membrane
FFA	Free Fatty Acids
GPK	Gas Phase Kinetics
GTD	Gas Transport Data File
MUFA	Mono Unsaturated Fatty Acids
PUFA	Poly Unsaturated Fatty Acids
SFA	Saturated Fatty Acids
TDF	Thermodynamic Data File
THC	Total Hydro-Carbons

## Data availability

The data will be made available on request**.**

## CRediT authorship contribution statement

**Valiveti Sivaramakrishna:** Writing – original draft, Visualization, Validation, Software, Resources, Methodology, Investigation, Formal analysis, Data curation, Conceptualization. **Shaik Hussain:** Visualization, Supervision, Software, Resources, Methodology, Investigation, Funding acquisition, Formal analysis, Conceptualization. **Chintalapudi Ravi Kiran:** Validation, Supervision, Software, Resources, Project administration, Methodology, Investigation, Funding acquisition, Formal analysis, Data curation. **Jayashri N. Nair:** Writing – review & editing, Writing – original draft, Visualization, Software, Resources, Project administration, Funding acquisition, Formal analysis, Data curation, Conceptualization. **Irfan Anjum Badruddin:** Visualization, Validation, Supervision, Software, Resources, Methodology, Investigation, Funding acquisition, Data curation, Conceptualization. **Abdul Saddique Shaik:** Visualization, Software, Resources, Methodology, Investigation, Formal analysis, Data curation, Conceptualization. **Sarfaraz Kamangar:** Validation, Software, Resources, Project administration, Methodology, Investigation, Funding acquisition, Data curation, Conceptualization. **Muhammad Mahmood Ali:** Writing – review & editing, Writing – original draft, Visualization, Resources, Project administration, Investigation, Funding acquisition, Formal analysis, Data curation, Conceptualization. **Muhammad Nasir Bashir:** Writing – review & editing, Writing – original draft, Visualization, Supervision, Project administration, Methodology, Investigation, Formal analysis, Data curation, Conceptualization.

## Declaration of competing interest

The authors declare that they have no known competing financial interests or personal relationships that could have appeared to influence the work reported in this paper.
